# Protective Effect of Cinnamon (*Cinnamomum zeylanicum)* Extract on the Sperm Quality During Cool Storage of Ram Semen

**DOI:** 10.1002/vms3.70370

**Published:** 2025-05-01

**Authors:** Reza Heidarzadeh, Saleh Tabatabaei Vakili

**Affiliations:** ^1^ Department of Animal Science, Faculty of Animal Science and Food Technology Agricultural Sciences and Natural Resources University of Khuzestan Mollasani Iran

**Keywords:** antioxidant, cinnamon, ram, semen quality

## Abstract

**Background:**

Oxidative stress, caused by an imbalance between reactive oxygen species (ROS) and antioxidants, poses a significant threat to sperm quality during storage.

**Objective:**

This study aimed to evaluate the protective effects of cinnamon extract, a natural antioxidant, on ram sperm quality during liquid storage at 4°C.

**Methods:**

Semen samples from 10 rams were collected, diluted and divided into five treatment groups. Each group received different concentrations of cinnamon extract (0, 50, 100, 150 and 200 µL/mL) added to the diluted semen. Sperm quality was evaluated at storage intervals of 1, 24, 48 and 72 h, with all samples maintained at 4°C during the storage period.

**Results:**

This study demonstrated that cinnamon extract significantly enhances ram sperm quality during liquid storage at 4°C. Notably, the addition of cinnamon extract, particularly at a concentration of 100 µL/mL, resulted in substantial improvements in progressive motility, total motility, sperm viability and plasma membrane integrity over the storage period. Specifically, after 1 h, the 50 µL/mL group showed a significant increase in progressive motility. At 24 h, both total and progressive motility were significantly higher in the 50, 100 and 150 µL/mL groups. By 48 h, the 50 and 100 µL/mL groups demonstrated significant enhancements in all measured parameters. The optimal results were observed at 72 h, where the 100 µL/mL group exhibited the highest values across all sperm quality metrics. These findings underscore the effectiveness of cinnamon extract as a natural antioxidant for preserving sperm quality during storage.

**Conclusions:**

Supplementation with cinnamon extract significantly enhances ram sperm quality, with an optimal concentration of 100 µL/mL effectively maintaining sperm viability during 72 h of liquid storage. These results suggest that cinnamon extract may serve as a valuable natural antioxidant for improving sperm preservation in liquid storage conditions.

## Graphical abstract

This study investigated the in vitro effects of cinnamon extract on sperm quality and viability during liquid storage in rams. The results showed that adding 100 µg/mL cinnamon extract to the diluent significantly improved ram sperm quality parameters during 72 h of storage at 4°C.

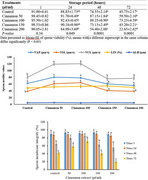

## 1 Introduction

The effective preservation of livestock semen is critical for maximizing the benefits of artificial insemination in animal breeding programmes. Semen storage typically involves refrigeration or freezing, methods that significantly reduce sperm metabolic activity and allow extended storage periods without a marked decrease in fertility (Bailey et al. 2000). While refrigeration maintains semen quality and mitigates complications associated with freezing, it is inherently limited by shorter storage durations, which can impact sperm viability and fertility retention (Hammerstedt et al. 1990; Forouzanfar et al. 2007).

Oxidative stress presents a major challenge to sperm integrity during storage. It arises from an imbalance between reactive oxygen species (ROS) production and the antioxidant defence mechanisms within the semen. Elevated ROS levels can damage critical cellular components, including proteins, lipids and DNA, ultimately compromising sperm function (Bansal and Bilaspuri 2010; Zargari 2020). Spermatozoa are particularly susceptible to oxidative damage due to their high content of unsaturated fatty acids in the plasma membrane and limited cytoplasmic defences, a result of substantial cytoplasmic loss during spermatogenesis (Agarwal and Prabakaran 2005).

Antioxidants play a pivotal role in protecting sperm from oxidative damage by neutralizing free radicals. These compounds can occur naturally in semen or be added synthetically and are essential for maintaining sperm viability during storage (Hamedani et al. 2013; Aslani and Ghobadi 2016). Numerous studies have shown that incorporating various antioxidants into semen diluents can significantly improve sperm motility, viability and acrosome integrity, thereby enhancing overall fertility potential (Hamedani et al. 2013).

In recent years, there has been an increasing interest in plant‐derived antioxidants, which can offer potent protective effects comparable to or even exceeding those of synthetic alternatives (Malo et al. 2011). Phytochemicals such as polyphenols, flavonoids and saponins are particularly effective at scavenging ROS and providing cellular protection against oxidative stress (Gülçin et al. 2012; Yazdi et al. 2019).

Cinnamon (*Cinnamomum zeylanicum*), a member of the Lauraceae family, is recognized for its diverse biological properties, including antioxidant, antibacterial and anticancer effects. The principal bioactive components in cinnamon, such as cinnamaldehyde and eugenol, exhibit strong antioxidant activities, functioning as effective reducing agents that can mitigate oxidative damage (Kumar et al. 2012; Yazdi et al. 2014; Rao and Gan 2014).

Despite the recognized antioxidant potential of cinnamon, its application in the preservation of semen, particularly in rams, remains somewhat unclear. Therefore, this study aims to investigate the effects of cinnamon extract on the viability and quality of chilled Arabi ram semen. We hypothesize that the incorporation of cinnamon extract will enhance sperm motility and overall quality during liquid storage, offering a novel approach to improve the preservation of ram semen.

## 2 Materials and Methods

### 2.1 Research Location and Animals

This study was conducted during the sheep breeding season using ten adult Arabian rams. The rams were 2.5 ± 0.3 years old and had an average weight of 70 ± 3.5 kg. They were housed at the farm animal research station of Khuzestan Agricultural Sciences and Natural Resources University, Iran. The rams had unrestricted access to water and food. Their diet consisted of a mixture of dry alfalfa (16%), barley (53%), wheat bran (14.5%), soybean meal (3%), oyster shell powder (0.5%), wheat straw (12%), salt (0.5%) and a mineral supplement (0.5%) with 2.53 Mcal/kg energy and 13% crude protein. Mineral lick blocks were also provided. This study was approved under the ethical approval number 1# 2022.4.24, Department of Animal Science, Faculty of Animal Science and Food Technology, Agricultural Sciences and Natural Resources University of Khuzestan, Mollasani, Khouzestan, Iran.

### 2.2 Preparation of Cinnamon Extract

*C. zeylanicum* barks were sourced from a herbal pharmacy shop in Ahwaz, Iran. The dried herbs were powdered in a mixer. 100 mL of the powder was combined with 900 mL of 96% ethanol, creating a 1:9 ratio. This mixture was shaken at room temperature for 24 h. After the extraction period, the mixture was strained through a clean screen cloth, followed by filter paper filtration to obtain a clear solution. The resulting liquid was concentrated using a rotary evaporator set at 40°C. Following concentration, the extracts were dried in an oven at 35°C for 24 h, effectively removing the ethanol. The final product was then stored at 4°C for preservation (Ariyan et al. 2021).

### 2.3 Sperm Collection, Semen Dilution and Experimental Treatments

Semen collection was performed weekly for 6 weeks using an electro‐ejaculator (Ogawa Seiki Co. Ltd., Japan). Electrical stimulation protocols were implemented, starting at a low voltage of 5 volts. The voltage was systematically increased in controlled increments up to 10 V. After each increment, the voltage was reset to zero, followed by a 10–20 s recovery interval. Each voltage was applied for a precise duration of 5 s. The threshold for physiological responses, specifically ejaculation, was assessed at voltage levels between 8 and 9 V. Semen samples were mixed to eliminate individual effects and diluted with a tris‐based extender (36.3 g tris, 5 g fructose, 20 g citric acid, 140 mL egg yolk and 1000 mL distilled water) (Salmon and Maxwell 2000). The average volume and sperm concentration of the rams were 1.35 ± 0.24 mL and 2.2 ± 0.18 billion/mL, respectively. The diluted semen was divided into five groups and received zero (control group), 50, 100, 150 and 200 µL/mL levels of cinnamon extract. The selection of cinnamon extract levels used in this research is somewhat based on the previous study on goats (Ariyan et al. 2021). The sperm quality parameters, including total and progressive motility, viability, plasma membrane integrity and morphological abnormalities, were evaluated at 1, 24, 48 and 72 h after storage in liquid condition at 4°C.

### 2.4 Evaluation of Sperm Quality Parameters

A total of 30 semen samples (6 weeks × 5 treatments) were subjected to sperm quality analysis. To assess sperm motility parameters, a minimum of 10 randomly selected microscopic fields from each sample were analysed using a computer‐assisted sperm analysis system (CASA; Video Test Sperm 2.1, Ltd., Russia). A total of 5 mL of seminal fluid was introduced into a counting chamber for evaluation. The following motility parameters were measured: total motility, progressive motility, average path velocity (VAP, µm/s), straight‐line velocity (VSL, µm/s), curvilinear velocity (VCL, µm/s), linearity (LIN, %), lateral head displacement (ALH, µm) and straightness of pass (STR, %) was calculated as VSL/VAP × 100. Additionally, the velocity rate of spermatozoa was categorized as rapid (VCL above 75 µm/s), medium (VCL between 45 and 75 µm/s) and slow (VCL below 45 µm/s), as described by Ba‐Awadh et al. (2023). All measurements were conducted at a magnification of 200×. Sperm viability and abnormalities were determined using the eosin–nigrosin staining (1.6 g eosin, 10 g nigrosin, 2.9 g sodium citrate and 100 mL distilled water). This involved mixing diluted semen with eosin–nigrosin stain in a 1:1 ratio, spreading the mixture on a slide and air‐drying. Slides were examined under a light microscope at 1000× magnification using immersion oil as described by Agarwal et al. (2022). The percentage of live and abnormal sperm was determined by counting at least 200 sperm in several fields of the slide. Live sperm appeared white, while dead sperm stained pink (Blom 1983; Evans and Maxwell 1987). Sperm cells exhibiting partial staining of the neck region, with the remainder of the head area remaining unstained, were classified as having a leaky neck membrane. Such sperm cells were considered alive, as the localized membrane compromise does not reflect complete membrane breakdown (Agarwal et al. 2022).

Plasma membrane integrity was evaluated using the hypo‐osmotic swelling test (HOST). A hypo‐osmotic solution (0.37 g sodium citrate dehydrate, 1.35 g fructose in 100 mL distilled water, pH 7) was prepared. A five‐microliter semen sample was mixed with 50 microliters of this solution and incubated at 37°C for 30 min. At least 200 sperm in five fields were examined under a light microscope at 400× magnification. Sperm with swollen and curled tails, indicating intact plasma membranes, were considered positive, while those without these characteristics were deemed negative (damaged plasma membrane) (Garcia‐Artiga 1994).

### 2.5 Statistical Analysis

Data were analysed using SPSS software (version 20) with a completely randomized design. Before conducting statistical tests, the normality of the data was assessed using the Shapiro–Wilk test. A one‐way analysis of variance (ANOVA) was conducted to compare sperm quality parameters among treatment groups at each storage time point. Repeated measures ANOVA was employed to assess changes in sperm parameters over time for each treatment group. Duncan's multiple range test was used for post hoc comparisons of means.

## 3 Results

### 3.1 Sperm Motility

**FIGURE 1:**
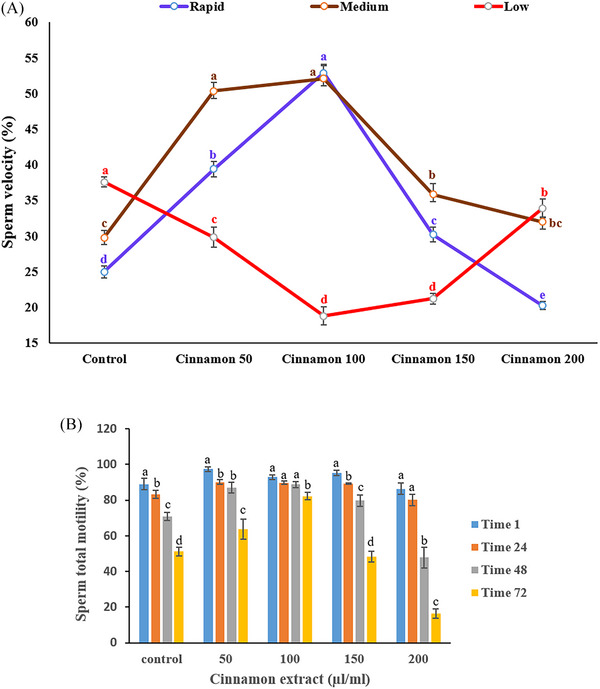
(A) Effect of different concentrations of cinnamon extract on sperm velocity 72 h after semen storage under chilled conditions in rams. Means with a different superscript in each parameter differ significantly (*p* < 0.05). The velocity rate of spermatozoa was classified as rapid (VCL greater than 75 µm/s), medium (VCL between 45 and 75 µm/s) and slow (VCL less than 45 µm/s. (B) Effect of various cinnamon extract levels on sperm total motility over storage time.

Table 1 summarizes the effects of different concentrations of cinnamon extract on total sperm motility over time. After 1 h of storage, no significant differences in total sperm motility were observed between the treatment groups. However, by 48 h, sperm motility was significantly higher in the 50 and 100 µL/mL cinnamon extract groups compared to the other groups (*p* < 0.05). The 100 µL/mL concentration was found to be the most effective for maintaining total sperm motility up to 72 h (*p* < 0.05). The changes in total sperm motility over time for each concentration of cinnamon extract are depicted in Figure 1. The 100 µL/mL group showed the highest motility, which remained relatively stable for up to 48 h, followed by a gradual decline thereafter.

**TABLE 1:** Effect of different concentrations of Cinnamon extract on sperm total motility (%) during chilled semen storage in rams.

Treatments (µL/mL)	Storage period (h)			
	1	24	48	72
Control	89.07 ± 3.16	83.20 ± 2.34^b^	70.83 ± 2.20^b^	51.25 ± 2.39^c^
Cinnamon 50	97.38 ± 1.23	90.21 ± 1.20^a^	86.90 ± 3.05^a^	63.75 ± 5.54^b^
Cinnamon 100	92.95 ± 1.26	89.75 ± 0.85^a^	88.82 ± 1.68^a^	82.18 ± 2.05^a^
Cinnamon 150	95.28 ± 1.60	89.25 ± 0.48^a^	79.75 ± 3.07^ab^	48.35 ± 3.15^c^
Cinnamon 200	86.38 ± 3.18	80.10 ± 3.11^b^	47.78 ± 5.85^c^	16.35 ± 2.53^d^
*p* value	0.25	0.011	0.0001	0.0001

*Note*: Data presented as mean ± SE. Means with a different superscript in the same column differ significantly (*p* < 0.05).

**FIGURE 2:**
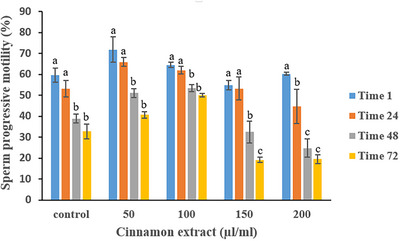
Effect of various cinnamon extract levels on sperm progressive motility over storage time.

Table 2 details the impact of cinnamon extract on progressive sperm motility. The 50 and 100 µL/mL groups exhibited the most effective preservation of progressive motility up to 48 h. At 72 h, the 100 µL/mL concentration again demonstrated superior efficacy in maintaining progressive motility (*p* < 0.05). Figure 2 illustrates the temporal changes in progressive sperm motility for each treatment group. The 50 and 100 µL/mL groups maintained relatively stable levels of progressive motility up to 48 h, with a significant decline observed at 72 h (*p* < 0.05).

**TABLE 2:** Effect of different concentrations of cinnamon extract on sperm progressive motility (%) during chilled semen storage in rams.

Treatments (µL/mL)	Storage period (h)			
	1	24	48	72
Control	59.60 ± 3.46^b^	53.25 ± 3.91^ab^	38.83 ± 2.09^b^	32.80 ± 3.46^c^
Cinnamon 50	71.78 ± 6.06^a^	65.93 ± 2.10^a^	51.15 ± 2.11^a^	40.62 ± 1.53^b^
Cinnamon 100	64.50 ± 1.19^ab^	61.98 ± 1.88^a^	53.45 ± 1.61^a^	50.10 ± 0.85^a^
Cinnamon 150	54.90 ± 2.26^b^	53.28 ± 5.41^ab^	32.50 ± 5.29^bc^	19.13 ± 1.31^d^
Cinnamon 200	60.33 ± 0.81^b^	44.60 ± 8.20^b^	24.83 ± 4.38^c^	19.50 ± 2.10^d^
*p* value	0.048	0.032	0.001	0.0001

*Note*: Data presented as mean ± SE. Means with a different superscript in the same column differ significantly (*p* < 0.05).

**FIGURE 3:**
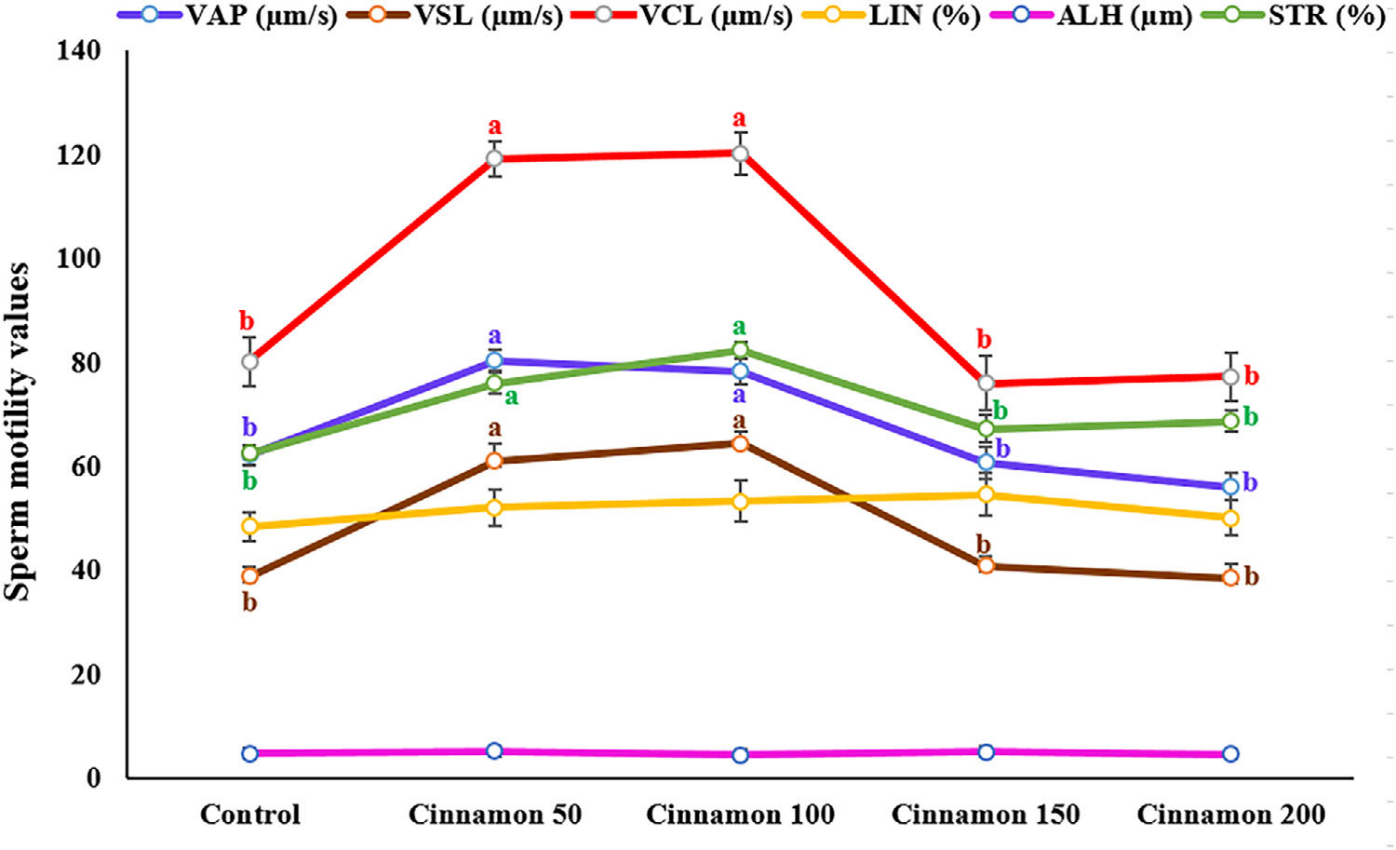
Effect of different concentrations of cinnamon extract on sperm motility parameters 72 h after semen storage under chilled conditions in rams. Means with a different superscript in each parameter differ significantly (*p* < 0.05).

Figure 3 illustrates the impact of cinnamon extract on detailed sperm motility parameters 72 h after semen storage. Spermatozoa treated with 50 µL/mL and 100 µL/mL cinnamon extract exhibited significantly higher values for VSL, VCL, VAP and STR than the other treatment groups (*p* < 0.05). In contrast, the treatments did not significantly affect the LIN and ALH parameters.

Figure 1 indicates the impact of cinnamon extract on sperm velocity ranges 72 h after semen storage. Spermatozoa treated with 100 µL/mL cinnamon extract exhibited significantly higher sperm rapid velocities than the control and other treatments. Additionally, the medium velocity was higher in the 50 and 100 µL/mL cinnamon extract groups (*p* < 0.05). The lowest sperm velocity was observed in the control group (*p* < 0.05).

### 3.2 Sperm Viability

**FIGURE 4:**
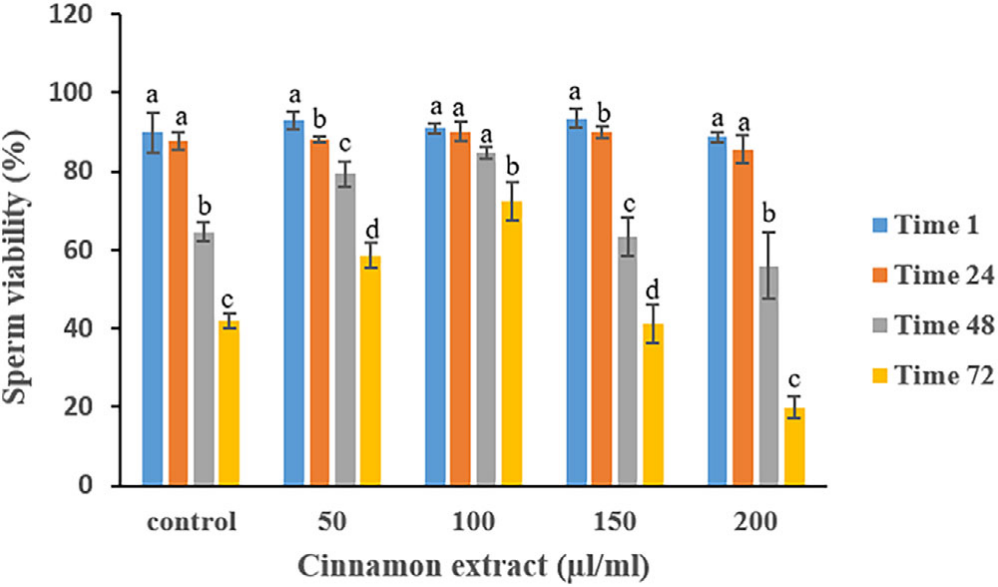
Effect of various cinnamon extract levels on sperm viability over storage time.

Table 3 shows the effects of cinnamon extract on sperm viability over time. At 1 h, no significant differences in sperm viability were observed between the treatment groups. However, at 48 h, the 50 µL/mL and 100 µL/mL cinnamon extract groups exhibited significantly higher sperm viability compared to the other treatments (*p* < 0.05). This pattern continued at 72 h, with the 100 µL/mL cinnamon extract group maintaining the highest sperm viability among all treatments, including the control (*p* < 0.05). In contrast, the 200 µL/mL cinnamon extract group consistently showed the lowest sperm viability throughout the storage period (*p* < 0.05). Figure 4 illustrates the time‐dependent changes in sperm viability for each treatment group. Although sperm viability declined over time for all groups, including the control and cinnamon extract treatments (*p* < 0.05), the rate of decline varied. The 100 µL/mL extract group exhibited the slowest rate of decline, suggesting a more effective preservation of sperm viability.

**TABLE 3:** Effect of different concentrations of cinnamon extract on sperm viability (%) during chilled semen storage in rams.

Treatments (µL/mL)	Storage period (h)			
	1	24	48	72
Control	91.00 ± 6.61	88.85 ± 1.73^ab^	74.55 ± 2.14^b^	45.75 ± 2.17^c^
Cinnamon 50	98.45 ± 0.82	91.70 ± 0.49^a^	87.15 ± 1.84^a^	59.50 ± 2.10^b^
Cinnamon 100	95.50 ± 1.02	92.43 ± 0.45^a^	89.25 ± 0.90^a^	73.25 ± 4.59^a^
Cinnamon 150	98.53 ± 0.86	90.18 ± 0.96^ab^	73.13 ± 2.49^b^	43.20 ± 2.21^c^
Cinnamon 200	90.05 ± 2.81	84.98 ± 3.69^b^	54.40 ± 2.08^c^	22.65 ± 2.82^d^
*p* value	0.34	0.049	0.0001	0.0001

*Note*: Data presented as mean ± SE. Means with a different superscript in the same column differ significantly (*p* < 0.05).

### 3.3 Sperm Plasma Membrane Integrity

**FIGURE 5:**
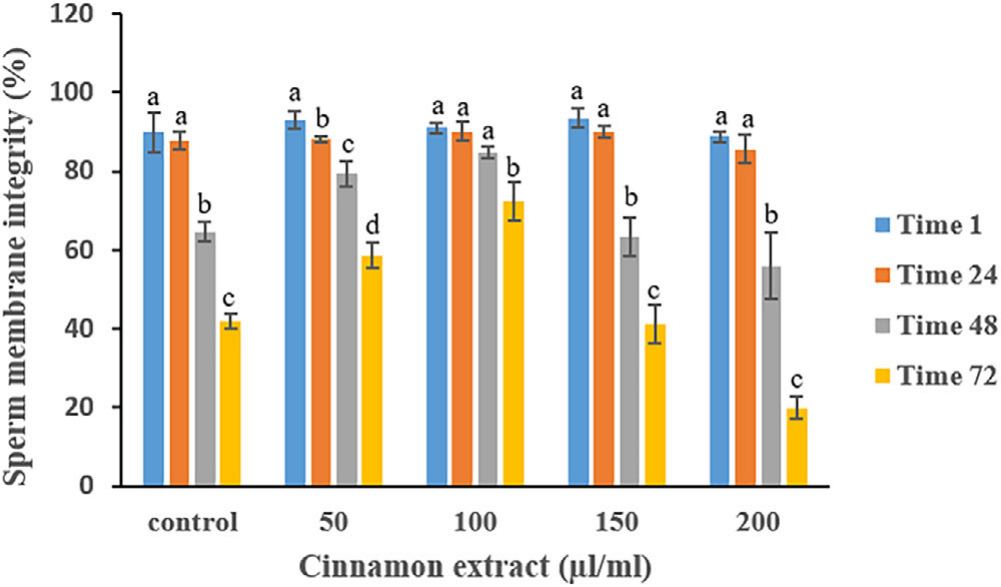
Effect of various cinnamon extract levels on sperm plasma membrane integrity over storage time.

Table 4 presents the effects of cinnamon extract on sperm plasma membrane integrity over time. No significant differences in plasma membrane integrity were observed among the treatment groups up to 24 h of storage. However, at 72 h, the 100 µL/mL cinnamon extract group demonstrated the highest plasma membrane integrity, with significantly higher values compared to all other treatments and the control group (*p* < 0.05). In contrast, the 200 µL/mL cinnamon extract group exhibited the lowest percentage of sperm with intact plasma membranes at this time point. As shown in Figure 5, while plasma membrane integrity declined in all groups over time, the 100 µL/mL cinnamon extract group demonstrated the slowest rate of decline compared to the control and the higher concentrations of cinnamon extract (*p* < 0.05).

**TABLE 4:** Effect of different concentrations of cinnamon extract on sperm membrane integrity (%) during chilled semen storage in rams.

Treatments (µL/mL)	Storage period (h)			
	1	24	48	72
Control	89.92 ± 4.98	87.65 ± 2.22	64.62 ± 2.44^b^	42.00 ± 1.78^c^
Cinnamon 50	92.95 ± 2.34	88.20 ± 0.84	79.43 ± 3.16^a^	58.65 ± 3.13^b^
Cinnamon 100	90.98 ± 1.32	90.08 ± 2.41	84.88 ± 1.45^a^	72.33 ± 4.98^a^
Cinnamon 150	93.53 ± 2.47	90.05 ± 1.43	63.35 ± 4.83^b^	41.18 ± 5.01^c^
Cinnamon 200	88.80 ± 1.38	85.53 ± 3.57	55.98 ± 8.34^b^	19.88 ± 2.82^d^
*p* value	0.24	0.61	0.022	0.0001

*Note*: Data presented as mean ± SE. Means with a different superscript in the same column differ significantly (*p* < 0.05).

### 3.4 Sperm Morphological Defect

**FIGURE 6:**
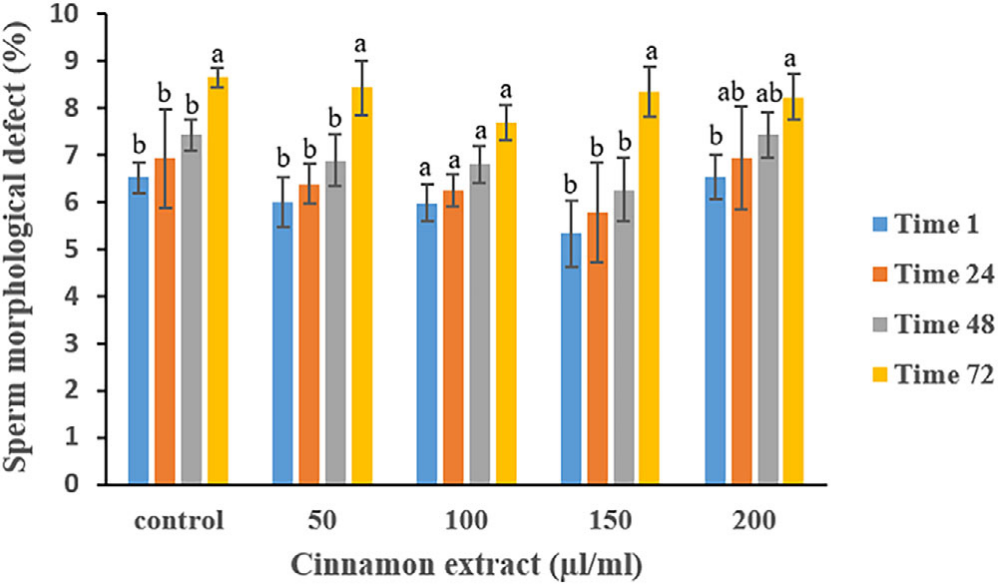
Effect of various cinnamon extract levels on sperm morphological abnormalities over storage time.

Table 5 presents the effects of cinnamon extract on sperm morphological abnormalities over time. No significant differences in sperm morphological abnormalities were observed among the treatment groups at any time point during semen storage. However, the 100 µL/mL cinnamon extract group maintained a significantly more stable percentage of normal sperm throughout the storage period compared to the control and other treatment groups (*p* < 0.05) (Figure 6).

**TABLE 5:** Effect of different concentrations of cinnamon extract on sperm morphological abnormalities (%) during chilled semen storage in rams.

Treatments (µL/mL)	Storage period (h)			
	1	24	48	72
Control	6.53 ± 0.33	6.93 ± 1.05	7.43 ± 0.33	8.65 ± 0.21
Cinnamon 50	6.00 ± 0.54	6.38 ± 0.42	6.88 ± 0.55	8.43 ± 0.57
Cinnamon 100	5.98 ± 0.38	6.25 ± 0.34	6.80 ± 0.38	7.68 ± 0.38
Cinnamon 150	5.33 ± 0.70	5.78 ± 1.05	6.26 ± 0.67	8.35 ± 0.52
Cinnamon 200	6.53 ± 0.48	6.93 ± 1.09	7.43 ± 0.48	8.23 ± 0.48
*p* value	0.46	0.52	0.48	0.63

*Note*: Data presented as mean ± SE. Means with a different superscript in the same column differ significantly (*p* < 0.05).

## 4 Discussion

The effects of cinnamon extract on sperm motility, viability, plasma membrane integrity and morphology were analysed across various concentrations and time points. The results indicate that cinnamon extract, particularly at 50 and 100 µL/mL concentrations, positively influenced several sperm parameters, notably sperm motility, viability and plasma membrane integrity. These findings are discussed for each parameter below, along with potential mechanisms underlying the observed effects.

### 4.1 Sperm Motility and Viability

The results from this study demonstrate that cinnamon extract significantly enhances sperm motility over time, particularly at the 50 and 100 µL/mL concentrations. By 48 h, both concentrations of cinnamon extract were more effective in maintaining total sperm motility than the control and lower concentrations, with the 100 µL/mL group maintaining the highest motility up to 72 h. These findings align with previous research suggesting that antioxidants, including those found in cinnamon, can improve sperm motility by reducing oxidative stress and protecting the testis from damage (Chegini et al. 2019). Cinnamon's antioxidant properties, attributed to compounds such as cinnamaldehyde, help mitigate oxidative stress, which has been shown to impair sperm motility (Yüce et al. 2013). The gradual decline in motility at 72 h in the 100 µL/mL group may reflect natural cellular senescence or oxidative stress over extended storage periods. However, the slower rate of motility decline compared to the control suggests that cinnamon extract may help preserve sperm function for longer periods by slowing oxidative damage (Arisha et al. 2020).

In the present study, sperm viability was significantly higher in the 50 and 100 µL/mL cinnamon extract groups compared to the control at both 48 and 72 h, with the 100 µL/mL concentration maintaining the highest viability throughout the storage period. These results suggest that cinnamon extract helps maintain sperm viability over time, likely due to its antioxidant properties, which help reduce the detrimental effects of ROS on sperm cells. Antioxidants like cinnamon work by scavenging ROS and preventing oxidative damage to critical cellular structures, such as mitochondria and the plasma membrane, thus supporting sperm survival (Kaltsas 2023). The 200 µL/mL cinnamon extract group, however, showed consistently lower sperm viability. This may indicate a potential toxic effect at higher concentrations, which has been observed with some natural extracts where excessive concentrations can overwhelm cellular protective mechanisms, leading to cytotoxicity (Lyoussi et al. 2018). A study by Ariyan et al. (2021) demonstrated that cinnamon extract enhanced sperm motility and viability in frozen‐thawed goat semen, corroborating our findings. Similarly, Yüce et al. (2013) reported feeding rats daily with 100 mg/kg/BW of cinnamon oil for 10 weeks increased testicular and epididymal weight and improved sperm motility. In another study, Khaki (2015) administered various doses of hydroalcoholic cinnamon extract to rats over 28 days and observed improvements in sperm quality parameters compared to the control group. Moreover, Pirami et al. (2018) found that administering 75 mg/kg of cinnamon extract to male rats with reproductive deficiencies enhanced sperm parameters, further supporting the role of cinnamon in reproductive health. Several studies have shown the positive effects of plant‐based antioxidants on sperm quality. Baghshahi et al. (2014) demonstrated that clove bud extract, similar to cinnamon, improved ram sperm motility post‐freeze‐thaw in a dose‐dependent manner. Similarly, the use of an extender containing 4%–6% rosemary aqueous extract rich in antioxidant compounds similar to those found in cinnamon improved motility, progressive motility and plasma membrane function in frozen‐thawed ram sperm (Gil et al. 2010). This suggests that antioxidants, such as those found in cinnamon, may protect sperm from oxidative stress and enhance their viability during storage.

### 4.2 Sperm Plasma Membrane Integrity and Morphology

The plasma membrane integrity of sperm was significantly preserved in the 100 µL/mL cinnamon extract group at 72 h, with the highest percentage of sperm showing intact membranes compared to other groups. This finding suggests that cinnamon extract helps preserve sperm membrane integrity, likely through its antioxidant effects. The integrity of the plasma membrane is critical for sperm functionality, and oxidative stress is known to damage lipid bilayers, leading to compromised membrane integrity (Wang et al. 2025). The slower decline in membrane integrity observed in the 100 µL/mL group may be due to cinnamon's ability to prevent lipid peroxidation, thereby protecting the sperm membrane from oxidative damage (Borzoei et al. 2018). Interestingly, the 200 µL/mL cinnamon extract group showed the lowest plasma membrane integrity, which further supports the idea that higher concentrations may have adverse effects on sperm health, possibly due to toxicity or overstimulation of antioxidant pathways (Walke et al. 2023). This suggests a concentration‐dependent effect, where moderate doses of cinnamon extract are beneficial, but higher doses could be harmful.

In the present study, no significant differences were observed in sperm morphological abnormalities across the treatment groups, although the 100 µL/mL cinnamon extract showed a more stable percentage of normal sperm throughout the storage period. This may indicate that cinnamon extract, at this concentration, provides some degree of protection against morphological defects, although this effect was not statistically significant. The maintenance of a higher percentage of normal sperm could be related to the antioxidant effects of cinnamon, which help reduce oxidative damage to sperm DNA and other cellular components involved in maintaining normal sperm morphology (Yüce et al. 2013). This aligns with previous findings suggesting that antioxidants protect sperm morphology in infertile men (Ahmadi et al. 2016). Furthermore, Allai et al. (2015) showed that adding lower concentrations of argan oil and cactus seed oil, both rich in antioxidants, to the semen extender improved plasma membrane integrity and morphology in ram sperm. These findings align with our study, indicating that lower levels of cinnamon extract positively affect sperm morphology parameters. Razavian et al. (2018) similarly found that adding 100 µL/mL of rose hip extract, which also possesses antioxidant properties, improved plasma membrane integrity compared to the control group, similar to the effects of cinnamon extract observed in this study. Other studies on the effects of sage extract and savoury extract on sperm quality have demonstrated improvements in membrane integrity following their addition to semen extenders, supporting the role of antioxidant‐rich plant extracts in preserving sperm quality during storage (Shahbazzadeh et al. 2015; Farhadi et al. 2016). In line with these findings, Asgari et al. (2020) showed that adding carob seed extract, which contains flavonoids and tocopherols, improved sperm quality in Farahani rams, further supporting the notion that antioxidants from plant extracts, including cinnamon, are effective in enhancing sperm quality. Hemmati et al. (2020) also found that adding a hydroalcoholic extract of Polygonum, another antioxidant‐rich plant, to rooster drinking water improved sperm plasma membrane integrity, further supporting the protective role of antioxidants in sperm preservation.

In our study, higher concentrations of cinnamon extract, particularly 200 µL/mL, had a detrimental effect on sperm quality. This is consistent with the findings of Malo et al. (2010), who reported that excessive antioxidant compounds at high concentrations can have a pro‐oxidant effect, leading to increased cellular damage. The dose‐dependent nature of the effect of cinnamon extract suggests that while moderate concentrations provide protective effects, higher concentrations may overwhelm the sperm's natural defence mechanisms, leading to oxidative stress and reduced sperm quality. Overall, this study supports using cinnamon extract as an effective agent for improving sperm quality during liquid storage in rams. The observed improvements can be attributed to the cinnamon's antioxidant and anti‐inflammatory properties, which help protect sperm from oxidative damage. These findings align with previous studies on the beneficial effects of plant‐derived antioxidants on sperm quality. However, further research is needed to better understand the mechanisms underlying these effects and optimize the concentration of cinnamon extract for sperm preservation in different species.

The improvements in sperm quality observed with cinnamon extract are likely due to its antioxidant and anti‐inflammatory properties, which help protect sperm from oxidative damage during storage. Cinnamon contains bioactive compounds such as cinnamaldehyde, which have been shown to reduce oxidative stress and improve sperm function. The cinnamon extract contains bioactive compounds such as cinnamaldehyde, which are known for their potent antioxidant properties. These compounds likely play a key role in the observed benefits to sperm motility, viability, membrane integrity and morphology. The antioxidant effects of cinnamon extract may act by scavenging ROS, which are known to cause oxidative damage to sperm cells. Furthermore, cinnamon extract may activate cellular defence mechanisms, such as the Nrf2/ARE pathway, which enhances the expression of antioxidant enzymes and helps protect sperm cells from oxidative stress (Rao and Gan 2014; Wondrak et al. 2010). Additionally, the preservation of sperm membrane integrity may be due to the protective effects of cinnamon extract on lipid peroxidation. Cinnamon has been shown to reduce lipid peroxidation in various cellular models, which may explain the higher sperm membrane integrity observed in the 100 µL/mL group (Soleimani et al. 2019). This antioxidant effect is particularly important, as sperm cells are highly vulnerable to oxidative damage, which affects their membrane function and overall fertility potential.

## 5 Conclusion

In conclusion, this study proves that cinnamon extract, particularly at 100 µL/mL, can effectively preserve sperm quality during liquid storage in Arabi rams. The observed improvements in motility, viability, membrane integrity and morphology can be attributed to the antioxidant and anti‐inflammatory properties of cinnamon. These effects highlight the potential of cinnamon as a natural preservative for semen storage. However, further research is needed to understand the exact mechanisms underlying these effects and to optimize the concentration of cinnamon extract for different species.

## Author Contributions

**Reza Heidarzadeh**: data curation, investigation, writing – original draft. **Saleh Tabatabaei**: methodology, formal analysis, supervision, writing – review and editing.

## Ethics Statement

The authors confirm that the ethical policies of the journal, as noted on the journal's author guidelines page, have been adhered to, and the appropriate ethical review committee approval has been received. The US National Research Council's guidelines for the Care and Use of Laboratory Animals were followed.

## Conflicts of Interest

The authors declare no conflicts of interest.

## Data Availability

The data that support the findings of this study are available on request from the corresponding author.
